# Methylseleninic acid restricts tumor growth in nude mice model of metastatic breast cancer probably via inhibiting angiopoietin-2

**DOI:** 10.1186/1471-2407-12-192

**Published:** 2012-05-28

**Authors:** Xiaojing Wu, Yidi Zhang, Zengyang Pei, Si Chen, Xu Yang, Yin Chen, Degui Lin, Runlin Z Ma

**Affiliations:** 1 The Clinical Department, College of Veterinary Medicine, China Agricultural University, Beijing, 100193, China; 2 State Key Laboratory of Molecular and Developmental Biology, Institute of Genetics and Developmental Biology, Chinese Academy of Sciences, Beijing, 100101, China; 3 Department of Pathology, Beijing Sanbo Brain Hospital, Beijing, 100093, China; 4 The Graduate University of the Chinese Academy of Sciences, Beijing, 100149, China

**Keywords:** Selenium, MSeA, Ang-2, VEGF, MDA-MB-231 cells, Xenograft tumor

## Abstract

**Background:**

Angiopoietin-2 (Ang-2) plays critical roles in vascular morphogenesis and its upregulation is frequently associated with various tumors. Previous studies showed that certain selenium compounds possess anti-tumor effects. However, the underlining mechanism has not been elucidated in detail. Plus, results of research on the anti-tumor effects of selenium compounds remain controversial.

**Methods:**

We investigated levels of Ang-2 and vascular endothelial growth factor (VEGF) on the estrogen-independent bone metastatic mammary cancer (MDA-MB-231) cells in response to treatment by methylseleninic acid (MSeA), and further examined the effects of MSeA oral administration on xenograft mammary tumors of athymic nude mice by RT-PCR, Western, radioimmuno assay, and Immunohistochemistry.

**Results:**

Treatment of MDA-MB-231 cells with MSeA caused significant reduction of *Ang-2* mRNA transcripts and secretion of Ang-2 proteins by the cells. Level of VEGF protein was accordingly decreased following the treatment. Compared with the controls, oral administration of MSeA (3 mg/kg/day for 18 days) to the nude mice carrying MDA-MB-231 induced tumors resulted in significant reduction in xenograft tumor volume and weights, significant decrease in microvascular density, and promotion of vascular normalization by increasing pericytes coverage. As expected, level of VEGF was also decreased in MSeA treated tumors.

**Conclusions:**

Our results point out that MSeA exerts its anti-tumor effects, at least in part, by inhibiting the Ang-2/Tie2 pathway, probably via inhibiting VEGF.

## Background

Selenium is an essential micronutrient element with a number of physiological functions in human. Trace amounts of Selenium are necessary for proper cellular function as components of the enzymes glutathione peroxidase and thioredoxin reductase, which indirectly reduce certain oxidized molecules in animals [1]. Selenium is also found in three deiodinase enzymes that convert one thyroid hormone to another [2]. A clear link between selenium and thyroid function was established, and optimal intake of selenium not only aids preservation of general health but also contributes substantially to the prevention of thyroid disease [3]. Several studies suggested possible links between cancer and selenium deficiency [4-7].

Results of researches on the anti-tumor effects of selenium compounds, however, remain controversial. Some studies showed selenium induced apoptosis and cell cycle arrest of prostate cancer cells *in vitro*[8-12]. Selenium decreased endothelial MMP-2 and VEGF in epithelial cancer cells, inhibited the mitosis and induced G1 arrest of umbilical vein endothelial [13-16]. Recent studies showed that MSeA treatment could downregulate hypoxia-inducible factor-1α in invasive prostate cancer [17]. At *in vivo* level, oral MSeA treatment of xenograft model animals inhibited the tumor growth in a dose-dependent manner [15,18-20]. Evidence indicates that dietary selenium altered prostate proteomic profiles, induced a set of tumor suppressor proteins [21] and prevented chemically-induced carcinogenesis in many rodent studies [22]. An earlier randomized, placebo-controlled clinical trial showed significant beneficial effects of selenium supplementation for cancer prevention in patients with skin carcinoma [23]. On the other hand, a randomized, placebo-controlled multi-centered clinical trial (Selenium and Vitamin E Cancer Prevention Trial, SELECT) of over 35,000 men showed selenium or vitamin E, alone or in combination at the doses and formulations used, did not prevent prostate cancer [24]. While the SELECT conclusion is convincing for the tested selenium compound of L-selenomethionine (SeMet), certain caution may be necessary in extrapolative interpretation of the results. Because ruling out the efficacy of SeMet does not necessarily ruling out the efficacy of all other bioactive Se forms [21,25]. Due to the documented differences in metabolic and biological properties between SeMet and MSeA, investigation on functions of MSeA still holds promise for potential cancer prevention.

Although previous studies showed certain selenium compounds possess anti-tumor effects, the underlining mechanism has not been fully elucidated. In particular, there is no detailed data showing exactly how MSeA is linked to inhibition of angiogenesis, a process critical for continuation of most tumor growth. Current evidences more or less linked to the inhibition of VEGF and MMP2 by MSeA, as well as to the G1 arrest of cells.

In this study, we investigated the effects of MSeA on the estrogen-independent bone metastatic mammary cancer (MDA-MB-231) cells and the tumors induced by the cells on athymic nude mice. We examined mRNA transcription and protein expression of Ang-2 at several concentration levels of MSeA on MDA-MB-231 cells, tested VEGF levels *in vitro* and *in vivo* in response to the MSeA treatments, compared the xenograft tumors for weight, volume and microvascular density following the MSeA treatment. Here we show direct evidence that MSeA at selected concentrations caused significant reduction in *Ang-2* mRNA transcription and Ang-2 protein secretion in MDA-MB-231 cells. Because Ang-2/Tie2 is known as a key regulator for tumor growth [26,27], our results help to explain the mechanism of anti-tumor function by MSeA.

## Results

### MSeA significantly inhibited Ang-2 secretion

**Figure 1:**
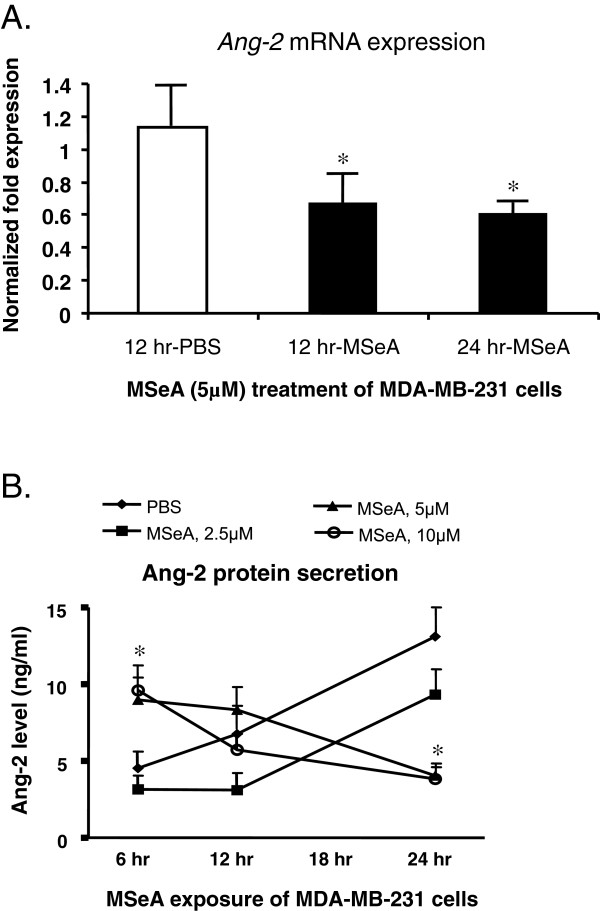
**Inhibition of *Ang-2 *mRNA transcripts and Ang-2 protein secretion in cultured mammary cancer cells by the MSeA treatment.** MDA-MB-231 cells were treated with selected concentrations of MSeA at the designated time intervals. Cellular mRNA and total protein from MSeA treated cells were isolated for subsequent quantitative analysis. **A**. Compared with the PBS controls, levels of *Ang-2* mRNA in MDA-MB-231 cancer cells were significantly reduced 12 hrs following the MSeA (5 μM) treatment, determined by quantitative RT-PCR (p < 0.05). MSeA treatment of the cells for 24 hrs did not further inhibit *Ang-2* mRNA expression. **B**. Secretion of Ang-2 protein into the medium by the MDA-MB-231 cells were significantly inhibited (p < 0.05) 24 hrs following the MSeA treatment at concentrations 5 μM or higher, as determined by Radioimmunoassay (RCA). Compared with the PBS control, a brief but significant increase in Ang-2 protein secretion was observed for the MDA-MB-231 cells at 6 hrs following the 5 μM and 10 μM MSeA treatments, and the reason is currently unknown to us.

Compared with the PBS-controls, treatment of the MDA-MB-231 mammary cancer cell cultures with MSeA at selected non-toxic concentrations (5 μM) significantly decreased *Ang-2* mRNA transcription at the designated time intervals (Figure 1A). The inhibitory MSeA on *Ang-2* mRNA was significant 12 h after the treatment (p < 0.05), and the prolonged treatment to 24 h did not further down regulate *Ang-2* mRNA expression.

Radioimmunoassay showed that the secretion of Ang-2 protein by MDA-MB-231 cells into the medium was significantly inhibited by MSeA in a concentration-dependent manner (Figure 1B), consistent with that of *Ang-2* mRNA transcription. The baseline secretion of Ang-2 (MSeA = 0 μM) showed a steady increase over time, and the treatment of MSeA at lower concentration (2.5 μM) did not significantly affect the trend of secretion. In contrast, higher concentrations of MSeA (≥5 μM) significantly reversed the levels of Ang-2 protein to the minimum 24 h following the treatment (p < 0.05). We also noticed a brief but significant increase in the level of Ang-2 protein at 6 hrs following the 5 μM and 10 μM MSeA treatments (Figure 1B), and the significance of this phenomenon is currently unknown to us.

### MSeA decreased level of VEGF production

**Figure 2:**
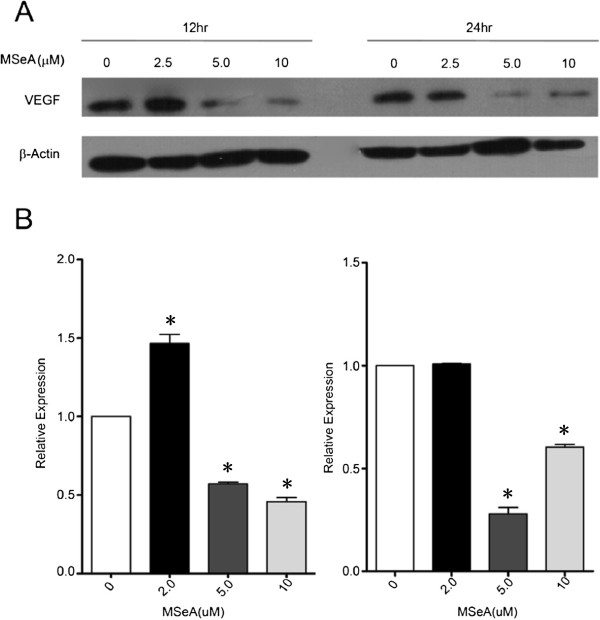
**Western blot showing the level of vascular endothelial growth factor (VEGF) in MDA-MB-231 cells was dramatically reduced following the treatment of MSeA at 5 μM for 12 hrs.** A higher MSeA concentration (10 μM) or longer treatment period (24 hrs) did not further inhibit the VEGF production. Commercial antihuman VEGF antibody (1: 200XD) was used to visualize the cellular VEGF protein band at the given MSeA concentrations and treatment time periods.

Western blot experiments showed that, comparing with the indigenous level of β-Actin, exposing MDA-MB-231 cells to MSeA treatment caused distinct reduction in the cellular VEGF protein in a concentration-dependent manner (Figure 2). MSeA at a lower concentration (2.5 μM) showed no significant effect on level of VEGF 12 h after the treatment. However, the inhibition effect was obvious at higher MSeA concentrations (5 μM and 10 μM). Prolonged MSeA treatment of the cells for 24 hrs did not further inhibit the VEGF protein production.

**Figure 3:**
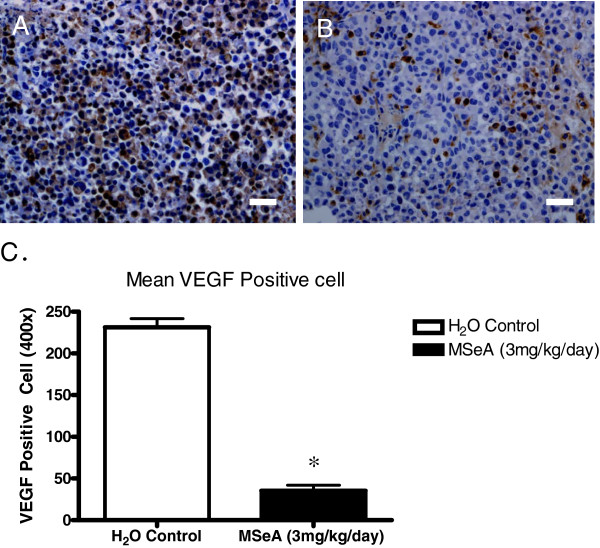
**Immunohistochemistry (IHC) stain for VEGF showed a lower level of VEGF expression in the xenograft tumor from the animal received MSeA-treatment. A.** Control. Target VEGF protein (brown cytoplasm) was extensively and highly expressed in a MDA-MB-231 cell-induced tumor of a representative nude mouse receiving no MSeA administration as control. **B.** MSeA treatment. VEGF protein expression was at a much lower percentage and level in xenograft tumor of a representative nude mouse receiving oral administration of MSeA at 3 mg/kg/day for 18 days. Scale bar = 50 μm. **C.** Statistical summary of VEGF positive cells for the control tumor (Mean = 234 cells/field, n = 3) and the MSeA treatment (Mean = 45 cells/field, n = 3) (P < 0.05).

Immunohistochemistry analysis of the tumor VEGF showed that the xenograft tumors of athymic nude mice induced by MDA-MB-231 cells had a distinct reduction of VEGF following the MSeA treatment (Figure 3). Oral administration of MSeA to the nude mice significantly reduced the number of cells producing VEGF, as visualized by the IHC staining (Figure 3B). The averaged intensity score of cellular VEGF for the MSeA-treated tumor was 1.0 (weak staining), while as the same score for the non-MSeA treated control tumor was greater than 2.0 (dark brown cytoplasm staining).

### MSeA restricted the growth of xenograft tumors

**Figure 4:**
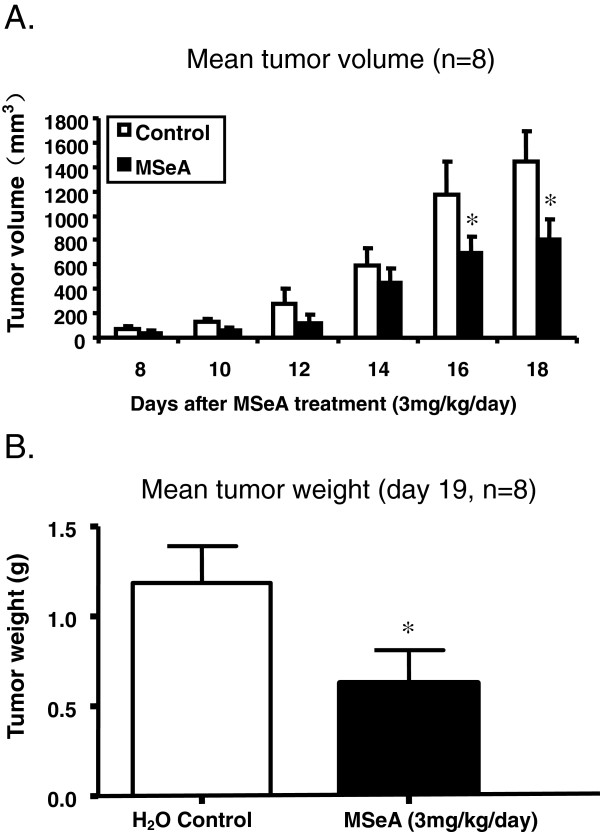
**Mean tumor volume (A) and tumor weight (B) of MDA-MB-231 mammary cancer cell induced xenografts were significantly lower (p < 0.05) in athymic nude mice receiving single dose oral administration of MSeA at 3 mg/kg/day.** Control animals received no oral administration of MSeA.

Compared with water blank controls, oral administration of MSeA to the nude mice at 3 mg/kg/day for 18 days significantly reduced the growth of xenograft tumors induced by MDA-MB-231 cells (Figure 4). Average tumor volume deceased approximately 44% in the MSeA treatment group (Figure 4A), and the mean tumor weight decreased approximately 46% (Figure 4B). The reduction in the tumor volume became significant at 16 days following the MSeA treatment. Meanwhile, differences in mean body weight of the nude mice was not significant between the MSeA-treatment (w =17.9±0.8 g; n = 8) and the control group (w =18.7±0.7 g; n = 8).

**Figure 5:**
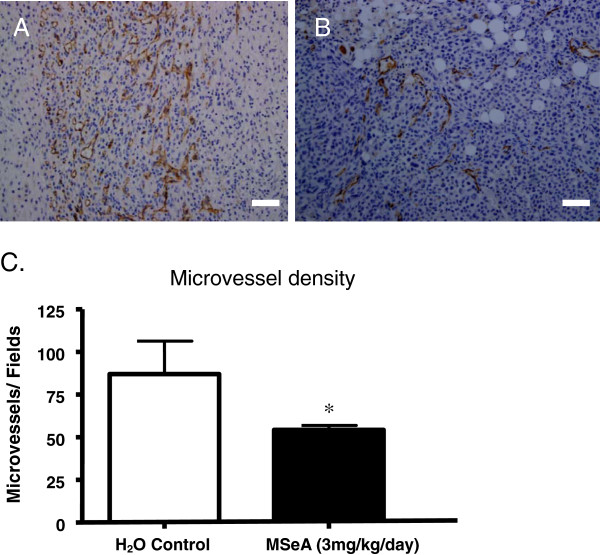
**MSeA treatment significantly reduced the microvessel density of the xenograft tumor. A.** Control. IHC staining for CD31 (brown) showed that the microvessel density was much higher in xenograft tumors from the nude mice received no oral administration of MSeA. **B.** MSeA treatment. Single dose oral administration of the nude mice with MSeA at 3 mg/kg/day x 18 days resulted in significant reduction in microvessel density, as indicated by CD31 IHC staining. Scale bar = 100 μm. **C.** Statistical summary of the microvessel density in the treatment and the control groups. A reduction of 38% in microvessel density was observed in the MSeA-treated nude mice (p < 0.05), with mean density for the control and MSeA-treatment group being 87±19 and 54±4, respectively (student-*t* test).

Immunohistochemistry (IHC) staining of the xenograft tumor sections using CD31 antibody showed that the microvessel density for the MSeA-treated tumor was significantly less (Figure 5B) when compared with that of non-MSeA controls (Figure 5A). Statistical analysis showed that the averaged microvessel density, expressed by the CD31-positive counting per 200X-view field for the MSeA-treatment and the control group, was 54±4 and 87±19, respectively. There was a 38% decrease in microvessel density in the treatment group (p < 0.05).

**Figure 6:**
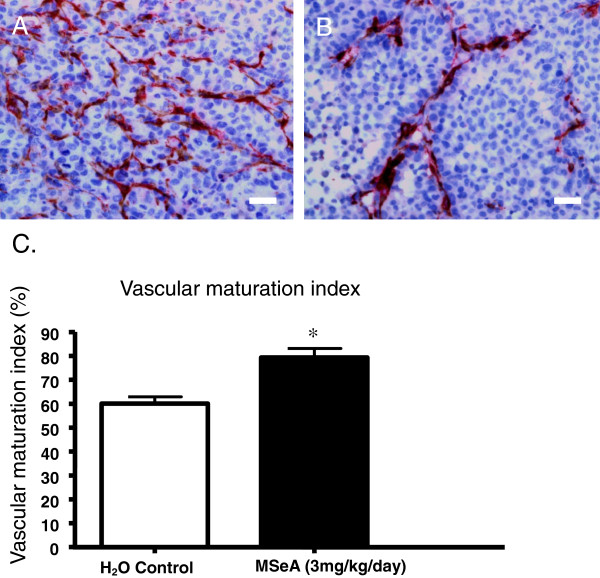
**MSeA facilitates vascular normalization by induction of vascular maturation. A.** Control. IHC double-staining for both of CD31and α-SMA showed that, the xenograft tumor from the nude mice with no MSeA treatment developed dense microvessels (red) but less surrounding pericytes (brown), indicating less maturation of the microvessels; **B.** MSeA treatment. The IHC double-staining showed increased presentation of α-SMA marker for pericytes (dark brown) along with the blood vessels of the tumor from the nude mice received oral administration of MSeA at 3 mg/kg/day for 18 days. Scale bar = 50 μm. **C.** Statistical summary of vascular density index. The Vascular Maturation Index (VMI) for the control group and the MSeA treatment group was 60±3% and 80 ±3%, respectively (p < 0.05)

MSeA induced microvascular maturation of the xenograft tumor. IHC double-staining using CD31 and α-SMA antibodies showed that, while the tumors from the control group exhibited more of CD31 staining for microvessel density (Figure 6A), the tumors receiving MSeA treatment showed more of α-SMA staining for pericytes (Figure 6B). The average percentage for α-SMA stain of the MSeA-treated and the control groups was 80±3% and 60±3%, respectively. Quantitative analysis of pericyte coverage showed about 20% increase in VMI (Vascular Maturation Index) in MSeA-treated tumors compared with the non-treated tumors (Table 6C, p < 0.05). Increased pericytes coverage is considered as a sign of microvascular maturation, which promotes vascular normalization.

**Figure 7:**
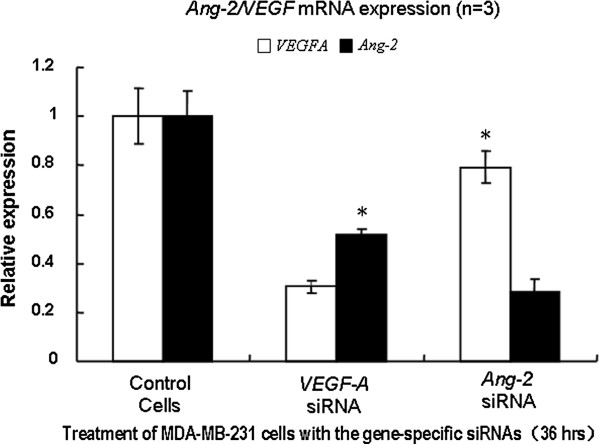
**RNA interference of *VEGF *and *Ang-2. ***MDA-MB-231 cells were transfected with the pooled siRNAs with lipofectamine 2000 (Invitrogen). Thirty-six hours after the transfection, total RNA from cultured cells was extracted by use of Trizol (Invitrogen). Real-time quantitative PCR was conducted to assess the level of the target mRNA expression using SYBR green dye, with relative changes calculated by the ΔΔCt method. While the suppression of either of *VEGF* or *Ang-2* caused significant reduction of the other when compared with the siRNA controls, inhibition of *VEGF* lead to a dramatic decrease in the level of *Ang-2*. The results indicate that *Ang-2* is more likely regulated by *VEGF*.

Suppression of endogenous *VEGF* or *Ang-2* mRNA expression by RNA interference (RNAi) in MDA-MB-231 cells showed that, while both of the levels of *VEGF* and *Ang-2* dropped significantly when any one of the two was inhibited, siRNA inhibition of *VEGF* caused dramatic drop of *Ang-2* expression (Figure 7). In contrast, siRNA inhibition of *Ang-2* caused less drop of *VEGF* level. Our results indicate that *Ang-2* was more likely located downstream of *VEGF*.

## Discussion

Our data showed that oral administration of MSeA to the athymic nude mice could significantly restrict the growth of xenograft tumors induced by mammary cancer MDA-MB-231 cells. Compared with the corresponding controls, the anti-tumor effects of MSeA were demonstrated in the nude mice model by significant reduction of xenograft tumor volume and weight, significant decrease in microvessel density of the tumors, plus promotion of vascular maturation and normalization by increasing pericytes coverage. The level of VEGF was significantly decreased in the tumors of the mice receiving the MSeA treatment. It is well known that VEGF plays an important role in solid tumor progression by stimulating microvessel growth, which in turn facilitates the oxygen and nutritional supply to tumors [27,28]. Our results are consistent with and are comparable to the anti-tumor effects of MSeA previously reported by other researchers [11,16,18,25,29]. The statement that proper concentrations of MSeA is potentially beneficial to human health in possessing the anti-tumor effects have the solid support of experimental evidences, at least in the xenograft animal models.

Our results indicate that partial inhibition of Ang-2 secretion may contribute, at least in part, to the mechanism of anti-tumor effects of MSeA. The levels of both cellular VEGF and Ang-2 secretion were significantly decreased following the MSeA treatment (Figures 1 &2). Our results not only confirmed the previous reports for inhibition of VEGF by MSeA [13,14], but also demonstrated that MSeA could significantly reduce Ang-2 protein secretion, at least in the mammary cancer cells. Ang-2 can be produced by both vascular endothelial cells and certain cancer cells in relatively low level [30]. The level of Ang-2 in the serum of the nude mouse carrying the xenograft tumors was not measured due to potential confounding of secretion by both the xenograft tumor cells and the microvessels of mice origin. Instead we used MDA-MB-231 cells to estimate the effect of MSeA on Ang-2 secretion. To our knowledge, this is the first time that MSeA is linked to inhibition of Ang-2, a critical components in angiogenesis pathway. Our data showed that inhibition of Ang-2 secretion was apparently via the inhibition of *Ang-2* mRNA transcription (Figure 1A).

Both of our results and previous research showed that MSeA inhibited the VEGF, which partially explain the mechanism of action of the MSeA [13,14]. Direct evidence on whether or not MSeA works simultaneously on both VEGF and Ang-2, or it works on sequential in a signal pathway, is currently non-conclusive to us. Since some research showed that Ang-2 are likely placed at the down stream of VEGF pathway and are regulated by VEGF [31,32], it is possible that the mechanism of anti-tumor by MSeA is to inhibit Ang-2, probably via inhibiting VEGF. For the relationships between VEGF and Ang-2, our results of the siRNA experiments are consistent with the findings by Zhang et al. [32], which support the statement that VEGF regulates the Ang-2 in the vascular endothelial cells.

Demonstration of Ang-2 inhibition by MSeA treatment has the profound significance. Ang-2 belongs to a family of growth factors that are critically involved in blood vessel formation during developmental and pathological angiogenesis [28,33-35]. The Ang-2/Tie-2 system acts as a vascular specific ligand/receptor system to control endothelial cell survival and vascular maturation [26,36-38]. Association between MSeA and Ang-2 inhibition, therefore, provide additional evidence of MSeA as a plausible candidate for certain caner prevention and treatment.

## Conclusions

Our results not only showed that MSeA significantly restricted xenograft tumor growth at the concentrations chosen, but also demonstrate that MSeA exerts its anti-tumor effects, at least in part, by inhibiting the Ang-2/Tie2 pathway, probably via inhibiting VEGF.

## Methods

### Animals and cells

Female Balb/c athymic nude mice of 6–7 week old were purchased from the Laboratory Animal Center, Cancer Institute, Chinese Academy of Medical Sciences. The colonies were maintained under the SPF condition in the same facilities for all of the experimental procedures. The animal usage protocols were approved in advance by both of Animal Care and Use Committees of the Chinese Academy of Medical Sciences and Chinese Agricultural University.

Cultural stock of estrogen-independent mammary cancer cells (MDA-MB-231 cells) was purchased from American Type Culture Collection (ATCC, HTB-26™). Working stock of the cell lines were maintained under standard DMEM media with 10% fetal bovine serum and 1‰ penicillin/streptomycin as antibiotics (Hyclone), in a 37 °C incubator with 5% high purity CO_2_.

### Chemicals and reagents

Methylseleninic acid (MSeA) used was obtained commercially (Sigma, #541281, CH_3_SeO_2_H, >95%). Stock solutions of MSeA at 0.5 mg/ml and 10 mM concentration were prepared in deionized distilled water and PBS solution, respectively; the stock solutions were filter-sterilized and stored in 1 ml aliquots under −70 °C for routine usage. Antibodies for CD31 (Santa Cruz, SC-1506), α-SMA (Abcam, ab-5694), and VEGF (Santa Cruz, SC-152) were obtained commercially and used following the instructions of the providers. All other chemicals and reagents used were at molecular biology grade.

### Generation of xenograft tumor model

To investigate the effect of MSeA treatment on tumor growth, an animal model was generated by induction of xenograft tumors using MDA-MB-231 cells in athymic nude mice. MDA-MB-231 cells (~3X10^6^ per animal) were subcutaneously inoculated into the right flank of each mouse and the animals were then randomly divided into two groups for comparison. One group received single-dose oral gavage administration of MSeA (3 mg/kg BW/animal) per day for 18 days (MSeA-treatment) and the other group received H_2_O as control. The induced tumors in both groups were measured by vernier calipers for volume (length × width^2^ × 0.5) at the designated time intervals. At the 19^th^ day, all the tumors were harvested for subsequent analysis on tumor weights, microvessel densities, and vascular maturation.

Microvessel density of the xenograft tumors was determined by immunohistochemistry (IHC) staining of the tumor sections using CD31 antibody specific for endothelial cells of microvessels [39,40]. For both MSeA-treatment and control group, microvessel density index was determined by counting CD31-marked endothelial cell clusters on three chosen fields of the highest density blood vessels at (200X) magnifying power. Quantifications were independently performed by two investigators for statistical analysis. VEGF antibody was used to visualize levels of VEGF in the xenograft tumors for treatment and control groups.

Effect of MSeA on vascular maturation of the xenografts was determined by IHC double staining of the tumor sections with both CD31 and α-SMA antibodies [18,41]. For the MSeA-treatment and the control group, visual counting of double-stained α-SMA/CD31 areas vs. the positive CD31-only tissue areas was performed. Each tumor section was examined for ten random fields under 400X magnification for statistical analysis. The blood vessels of the MSeA treatment groups and the control group were analyzed independently by two investigators.

### Real-time PCR and Western blot

To assess whether MSeA could affect the transcriptional levels of key genes/mRNAs potentially associated with tumor development in MDA-MB-231 cells, total cellular RNA from the MSeA-treated and the PBS control cells were isolated using Trizol reagent (Invitrogen) [42]. For the treatment, MSeA at a concentration of 5 μM as added into the DMEM medium when cells reached 60-70% confluence. Control cells received PBS treatment. Total RNAs were isolated at 12 h and 24 h after the treatment and 2 mg of total RNA from each group was subjected for the 1^st^ strand cDNA synthesis by reverse transcription. Quantitative PCRs were performed with the SYBR Premix TaqReal-Time PCR Detection System (TaKaRa) on a real-time thermal cycler (Bio-Rad Laboratories, Inc., USA). Each qPCR reaction was run in triplicates and relative amount of mRNA for each gene was normalized based on house keeping gene *β-Actin*. The forward and reverse primer sequence for *Ang-2* mRNA amplification was 5^’^-AGATTTTGGACCAGACCAGTGA-3’ and 5’-GGATGATGTGCTTGTCTTCCAT-3’  respectively [22]. Total cellular proteins were isolated from both MSeA–treated and PBS-treated cells for Western analysis, and VEGF antibody was used to visualize level of cellular VEGF after electrophoresis.

### Radioimmuno assay

To examine whether the MSeA treatment could affect secretion of Ang-2 by MDA-MB-231 cells, radioimmuno assay was utilized to measure the level of Ang-2 in cell medium using a commercial Ang-2 RIA KIT (Sinoukbio, HY174). Instructions from the kit provider were followed in the entire experimental procedures and the data analysis. Detection principle is essentially the same as to that described previously [43]. Briefly, following the treatment of cells with MSeA at 0 μM (no treatment), 2.5 μM, 5.0 μM and 10 μM concentrations after 6 h, 12 h, and 24 h period of time, 50 μl of cell culture supernatant from each treatment was collected to measure the level of radioactivity-labeled ^125^I-Ang-2 in the medium on an R-911 full-automatic radioimmuno calculating instrument. Measurement for each treatment was conducted in triplicates.

### RNA interference of VEGF and ang-2

To assess the regulation relationships between VEGF and Ang-2 in MDA-MB-231 cells, RNA interference was utilized to suppress the target gene expression, and mRNA levels after the RNA interference were measured by the real-time quantitative PCR. Three sequence-specific siRNAs were designed for each of the target genes to ensure the effective suppression (Additional file 1: Table S1). MDA-MB-231 cells were transfected with the pooled siRNAs with lipofectamine 2000 (Invitrogen). Thirty-six hours after transfection, total RNA from cultured cells was extracted by use of Trizol (Invitrogen). Real-time quantitative PCR was conducted using SYBR green dye, with relative changes calculated by the ΔΔCt method.

### Statistical analysis

All the numerical data collected in the experiments were statistically analyzed by ANOVA or the student *t*-test between the treatments and the controls when applicable. The data were considered significant at p < 0.05.

## Competing interests

Authors declare no financial or non-financial competing interests.

## Authors’ contributions

XW and YZ carried out the molecular genetic studies, collected the data, and helped to draft the manuscript. ZP participated in the study design and performed the statistical analysis. SC participated in the data collection and helped to draft the manuscript. XY carried out some of the cell cultures. YC carried out the immunoassays. DL and RM designed the experiments, supervised the study, and wrote the manuscript. All authors read and approved the final manuscript.

## Pre-publication history

The pre-publication history for this paper can be accessed here:

http://www.biomedcentral.com/1471-2407/12/192/prepub

## Supplementary Material

Additional file 1 **Table S1.** cDNA sequences of *VEGF* and *Ang-2* siRNAs. Click here for file
