# Supplementary material for: Methylseleninic acid restricts tumor growth in nude mice model of metastatic breast cancer probably via inhibiting angiopoietin-2
Source: BMC Cancer. 2012 May 28;12:192. doi: 10.1186/1471-2407-12-192 (PMC3517305; doi:10.1186/1471-2407-12-192)
Supplement: Additional file 1 — Table S1. cDNA sequences of VEGF and Ang-2 siRNAs. [file 1471-2407-12-192-S1.doc]

| Target Gene | siRNA Name | Sequence | | |
| --- | --- | --- | --- | --- |
| *Ang-2* | Ang2-743 | sense | 5'-CGCGGAAGUUAACUGAUGUTT-3' | |
|  |  | anti-sense | 5'-ACAUCAGUUAACUUCCGCGTT-3' | |
|  | Ang2-853 | sense | 5'-GACCAGACCAGUGAAAUAATT-3' | |
|  |  | anti-sense | 5'-UUAUUUCACUGGUCUGGUCTT-3' | |
|  | Ang2-1425 | sense | 5'-GCAACGCUAUGUGCUUAAATT-3' | |
|  |  | anti-sense | 5'-UUUAAGCACAUAGCGUUGCTT-3' | |
| *VEGF* | VEGFA-1029 | sense | 5'-CCGAAACCAUGAACUUUCUTT-3' | |
|  |  | anti-sense | 5'-AGAAAGUUCAUGGUUUCGGTT-3' | |
|  | VEGFA-1137 | sense | 5'-GGCAGAAUCAUCACGAAGUTT-3' | |
|  |  | anti-sense | 5'-ACUUCGUGAUGAUUCUGCCTT-3' | |
|  | VEGFA-1398 | sense | 5'-GCUUCCUACAGCACAACAATT-3' | |
|  |  | anti-sense | 5'-UUGUUGUGCUGUAGGAAGCTT-3' | |
| *GAPDH* |  | sense | 5'-GUAUGACAACAGCCUCAAGTT-3' | |
|  |  | anti-sense | | 5'-CUUGAGGCUGUUGUCAUACTT-3' |

Table S1. The sense and anti-sense sequences of siRNAs used for suppression of the target gene
